# Nurse Communication About Goals of Care

**DOI:** 10.6004/jadpro.2016.7.2.2

**Published:** 2016-03-01

**Authors:** Elaine Wittenberg, Betty Ferrell, Joy Goldsmith, Haley Buller, Tammy Neiman

**Affiliations:** From 1 City of Hope National Medical Center, Division of Nursing Research and Education, Duarte, California;; 2 University of Memphis, Tennessee;; 3 Chapman University, Orange, California;; 4 St. Catherine University, St. Paul, Minnesota

## Abstract

Conversations about goals of care with the patient and family are a critical component of advanced practice in oncology. However, there are often inadequate team structures, training, or resources available to assist advanced practitioners in initiating these conversations. We conducted a study to assess nurses’ perceived role and communication tasks in such conversations about goals of care. In a cross-sectional survey of 109 nurses attending a comprehensive 2-day end-of-life nursing education course, nurses were asked to describe how they would participate in a "goals of care" meeting in three different scenarios. They were also asked what changes they desired in their clinical settings. Nurses overwhelmingly described that their primary task and communication role was to assess patient/family understanding. Nurses referenced their team members and team support with the least frequency across scenarios. Team roles, structure, and process were reported as areas in greatest need of change in patient/family goals of care meetings. These findings demonstrate that lack of preparation to function as a team is a barrier for nurses in communicating about goals of care, and there is a demand to move such conversations upstream in oncology care.

A difficult conversation has been defined as an interaction between aprovider and a patient at transition points on the disease trajectory (Svarovsky, 2013). In oncology, these transition points include sharing a new cancer diagnosis, deciding on treatment options, transitioning to survivorship, or shifting the focus to palliative care. These conversations, commonly known as "goals-of-care" discussions, often involve family and provide a platform for discussing and setting goals of care and sustaining hope by defining and reevaluating goals as the patient’s disease progresses (Svarovsky, 2013). The conversations should trigger early discussions, educate the patient and family, be documented in the electronic medical record, and be evaluated based on performance standards (Bernacki, Block, & American College of Physicians High Value Care Task Force, 2014).

Although goals-of-care conversations need to include trained advanced practitioners (APs) and target patients at high risk of death within the year, few APs have received formal communication training about these discussions, and hospital systems rarely have processes in place to ensure that these conversations take place. As a result, conversations about goals of care often occur too late to make a difference in the quality of care provided, patients are infrequently encouraged to actively participate in their own decision-making process, and often consent from the patient is implicit (Bélanger, Rodriguez, & Groleau, 2011). This article summarizes the literature on goals-of-care conversations and presents a study on nurses’ perception of these conversations.

## REVIEW OF THE LITERATURE

Goals-of-care conversations often do not occur due to patient/family, provider, and system barriers. Patient/family barriers include difficulty accepting a poor prognosis, difficulty in understanding complications of life-sustaining treatments, disagreement among family members, and the patient’s incapacity to participate in these important discussions (You et al., 2015). Provider barriers include insufficient knowledge about the patient, inconsistent assessment of the patient’s nonmedical goals, and failure to provide sufficient information for decision-making (Bernacki et al., 2014). Systemic barriers include obstacles to team communication, especially for sharing information across services and between specialties, with variation in documentation systems delaying workflow and care coordination (McCorkle et al., 2012). Advanced practitioners experience role tension due to the variability across disease teams and between inpatient and outpatient settings, which can create a disparity in team communication and impede coordination (McCorkle et al., 2012).

Despite these barriers, goals-of-care conversations remain a gold standard in facilitating patient-centered communication and shared decision-making (Bakitas, Kryworuchko, Matlock, & Volandes, 2011; Tamburro, Shaffer, Hahnlen, Felker, & Ceneviva, 2011). Shared decision-making is a process often characterized by patient-family-provider involvement, information sharing between parties, acknowledgment of all preferences, and agreement over future plans (Charles, Gafni, & Whelan, 1997; Moumjid, Gafni, Brémond, & Carrère, 2007). Shared decision-making is inhibited by unmet information needs and unrealistic expectations, the framing of options in consultations, and the patient’s/family’s wish to delay decisions to follow default patterns of care (Bélanger et al., 2011).

Although little is known about the impact of participation in decision-making, researchers have found that lower levels of health literacy have the potential to negatively influence patient participation (McCaffery et al., 2013). Health literacy is defined as an individual’s capacity to obtain, process, and understand basic health information and is often regarded as a highly important factor in patient-provider interactions (The Patient Protection and Affordable Care Act, 2010).

However, those individuals who are less health-literate often have more issues relating to successful patient-provider interactions. For instance, lower health literacy is associated with lower patient health knowledge (McCaffery et al., 2013) and with higher decisional uncertainty and regret. Low patient health knowledge and decisional uncertainty, if overlooked by the health-care team, can result in negative consequences such as miscommunication (Kawachi & Kennedy, 1997). In addition, individuals with lower health literacy often have less desire for involvement, ask fewer questions, and experience less patient-centered care (McCaffery et al., 2013). All of these factors could potentially influence patient and family participation in the shared decision-making process, which can be detrimental to the overall quality of any goals-of-care conversations.

Decision support tools such as brochures, audiovisual materials, educational sessions, counseling sessions, and interactive websites or media have been implemented to improve goals-of-care conversations and encourage participation in decision-making (Wu, Boushey, Potter, & Stacey, 2014); however, most of these tools are patient-focused rather than patient- and family-focused. This is problematic given the increasing reliance on family caregivers for assistance with care, transportation to appointments, and involvement in decision-making as proxy. Although family involvement is not necessary in every case, many families have reported feeling misunderstood or neglected when their involvement in the patient’s care is not considered (Elwyn et al., 2013b).

Although support tools have been developed, most of the patient decision aids are for independent use either before or after clinical visits (Elwyn et al., 2013a), and widespread adoption of these decision-support interventions has not yet occurred (Elwyn et al., 2013b). Further, few of these tools include decision-coaching, and many neglect to assess communication in the encounter (Elwyn et al., 2013b). Most decision tools are never fully implemented by providers due to time pressures, and providers often perceive a lack of applicability to patient characteristics or clinical situations (Wu et al., 2014).

Research and communication skills training about goals-of-care conversations are also heavily physician-focused (Svarovsky, 2013), neglecting the role of the AP (Elwyn et al., 2013b). Although patients do not express uniform agreement about which clinician they prefer for goals-of-care conversations (Bernacki et al., 2014), a variety of health-care providers have concluded that advanced practice nurses or social workers would be excellent providers for initiating such discussions and decision-coaching (You et al., 2015). Advanced practice nurses are expected to support decision-making by providing information, conveying empathy and building trust, and advocating to others about the patient’s wishes (Adams, Bailey, Anderson, & Docherty, 2011), yet there is little instruction available for them about how to communicate in a way that accomplishes this approach (Elwyn, Frosch, Volandes, Edwards, & Montori, 2010).

Nurses are expected to consider the patient’s values and quality-of-life preferences and to review them with the patient and family in a manner separate from their own daily goals and treatment tasks (Martin & Koesel, 2010). Given the emphasis on nurse communication skills to determine the goals and preferences of patients and families and assist with health-care decision-making (National Consensus Project for Quality Palliative Care, 2013), this study explored nurses’ perception of their role and communication tasks during goals-of-care conversations with patients and family in three different contexts.

## METHODS

An open-ended survey was distributed to nurses attending one of four End-of-Life Nursing Education Consortium (ELNEC) programs. These ELNEC programs are delivered in a 2-day train-the-trainer format, providing participants with comprehensive curriculum about end-of-life care including communication. Nurses voluntarily completed the survey prior to receiving course content. The survey was determined to be exempt under the institutional review board at the supporting institution.

**Instrument**

Consisting of four open-ended items, the survey was developed by nursing, communication, and oncology research experts. Nurses were presented with three different oncology case scenarios and asked to describe how they would participate in a goals-of-care meeting involving (1) the health-care team, patient, and family; (2) a patient, following physician disclosure of life-altering information; and (3) when a patient asks to talk about treatment decisions (Table 1). To assess areas for improvement, a final open-ended question invited nurses to describe what changes were needed for goals-of-care conversations in their current position or setting.

**Table 1:**
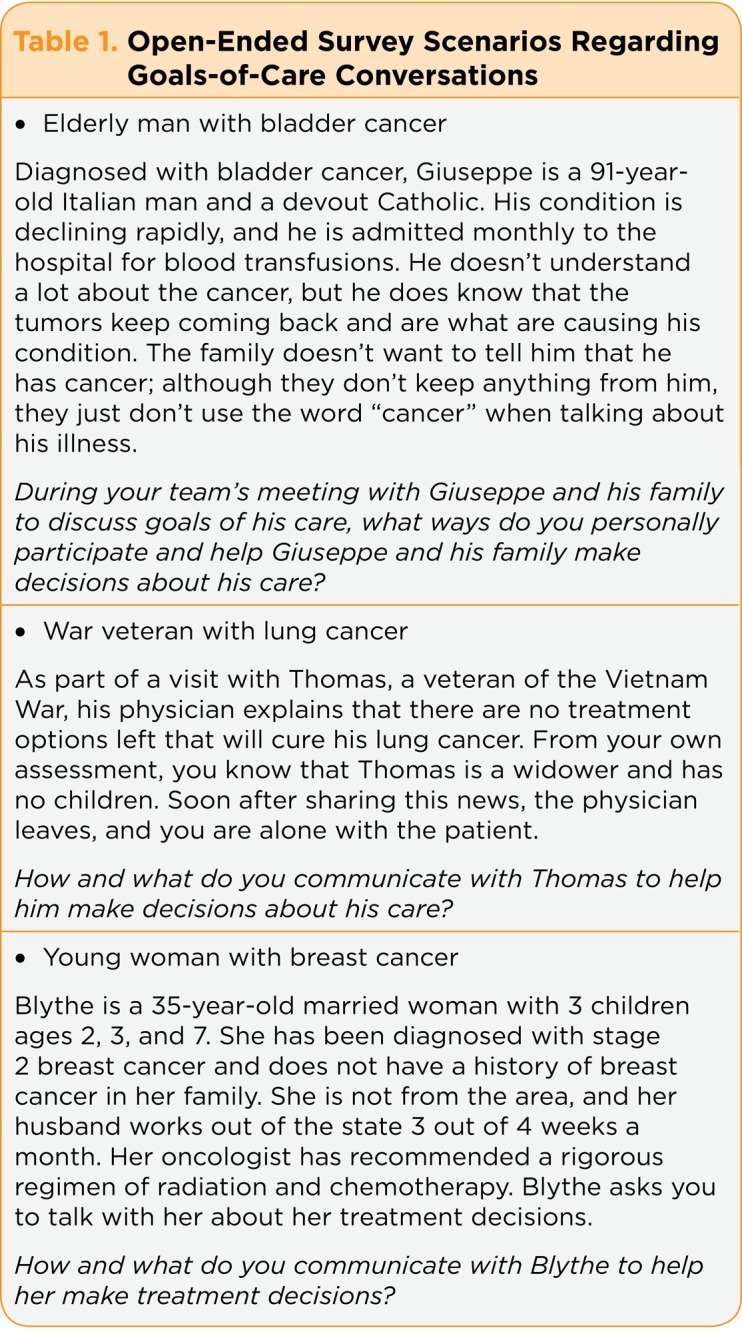
Open-Ended Survey Scenarios Regarding Goals-of-Care Conversations

**Data Analysis**

A research team member transcribed all open-ended responses, and three members of the research team reviewed them. Inductive-content analysis was used in two phases (Elo & Kyngäs, 2008). First, each research team member individually conducted open coding by identifying unrestricted chunks of text and creating categories. Second, research team members met to integrate categories and come to consensus by discussing independently created identified themes and connecting, collapsing, or associating to establish categories. Identification of categories emerged from strong representation throughout responses and was verified by coding and frequency calculation.

## RESULTS

**Study Participants**

A total of 193 nurses were surveyed, with 109 completing the open-ended items of the survey. Half of the nurses surveyed had more than 16 years of nursing experience. They came from a variety of settings, most commonly hospital (50%) and university (25%) settings. Table 2 summarizes the participant demographics.

**Table 2:**
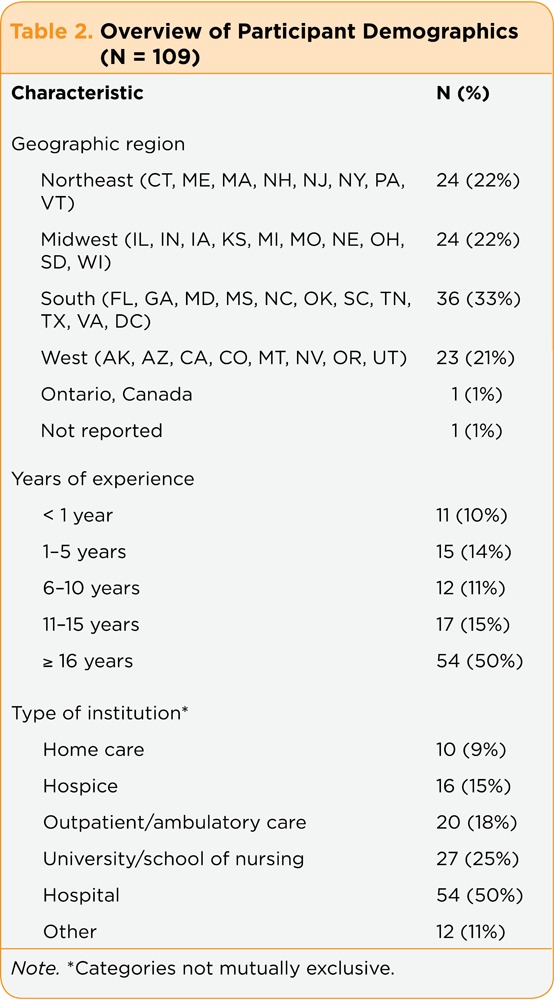
Overview of Participant Demographics (N = 109)

Participants overwhelmingly described assessing patient understanding as the dominant goal-of-care communication. Across all three scenarios, the nurses’ primary response was first to assess patient and/or family understanding of the disease and prognosis and ask questions about the patient’s goals of care.

The highest frequency of this communication task (n = 65) appeared in response to the third scenario, in which a patient requested a discussion with the nurse about treatment decisions. The second most common communication task/role in each of the three scenarios reflected the circumstances of each scenario.

The first scenario depicted team members present and nurses focusing attention on family (n = 42) as their key role (Table 3). In scenario two, following physician disclosure of a poor prognosis, nurses emphasized being present with the patient (Table 4) by describing listening (n = 56). In the third scenario, when asked by the patient about treatment decisions, nurses described inquiring about patient resources concerning care, side-effect tolerance, and place of care (n = 57) and reported that they would assess for understanding and also convey information honestly (Table 5).

**Table 3:**
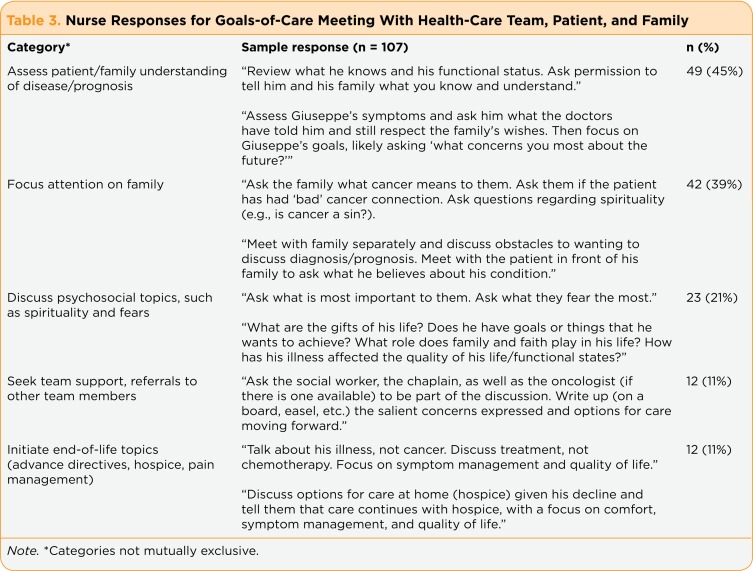
Nurse Responses for Goals-of-Care Meeting With Health-Care Team, Patient, and Family

**Table 4:**
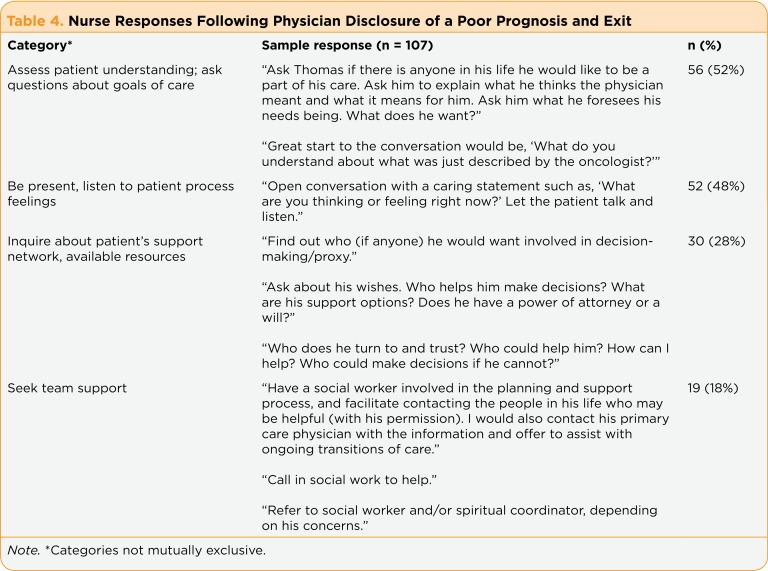
Nurse Responses Following Physician Disclosure of a Poor Prognosis and Exit

**Table 5:**
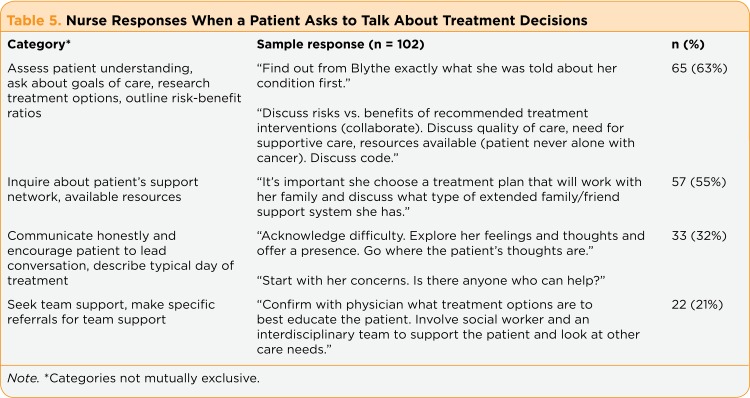
Nurse Responses When a Patient Asks to Talk About Treatment Decisions

Overall, participant comments showed little difference in approach to goals-of-care conversations and communicative tasks, despite the different scenarios presented. In none of the three scenarios were team members or support from other disciplines identified frequently, and in fact team members and team support was the least mentioned communication task/role element across all scenarios.

Finally, an open-ended question offered nurses an opportunity to share what they wished was different about goals-of-care communication in their work setting. In terms of needing change, nurses overwhelmingly pointed to team roles, structures, and processes as the areas of change needed to improve such communication (Table 6). These two findings (low frequency in scenarios and high frequency in desired changes) are in consonance with one another. Closely related to team structures and processes, nurses commonly described the need and desire for goals-of-care conversations to occur earlier in the cancer care continuum.

**Table 6:**
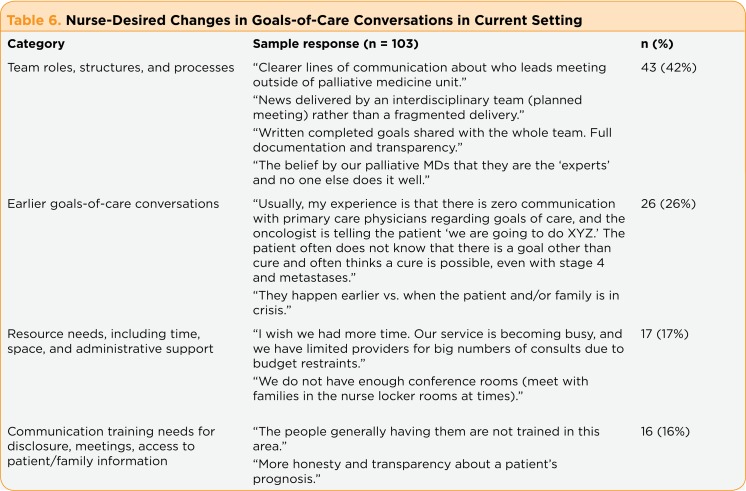
Nurse-Desired Changes in Goals-of-Care Conversations in Current Setting

## DISCUSSION

This study provides unique findings about nurse perceptions of their role and communication tasks during goals-of-care conversations across three oncology care scenarios. Additionally, nurses identified desired changes in goals-of-care communication in their own settings. As the sample in this study had extensive clinical experience, their task and role perceptions as well as challenges represent experiential credibility, strengthening these findings.

Overall, the dominating nurse response to goals-of-care tasks was representative of nursing education to assess patient understanding. Recent work has shown that years of nursing experience is positively associated with comfort in communicating about end of life, one of the transition points in a goals-of-care discussion (Moir, Roberts, Martz, Perry, & Tivis, 2015).

Still, nurses experience unclear team roles when providing palliative care to patients/families (Beckstrand, Collette, Callister, & Luthy, 2012; Klarare, Hagelin, Fürst, & Fossum, 2013). Palliative care communication is a unique skill that nurses engage in across care settings and throughout the disease trajectory (Goldsmith, Ferrell, Wittenberg Lyles, & Ragan, 2013; Malloy, Virani, Kelly, & Munevar, 2010). There is little question that nurses have distinctive needs and demands in their goals-of-care communication and should be educated to handle the communication demands placed upon them.

Although assessing patient understanding is a key focus of goals-of-care conversations, there is also a general consensus that these conversations should include providing the prognosis and discussing acceptable trade-offs and function for the patient (Bernacki et al., 2014). Patient preferences for goals of care often include statements about length of life, symptom control, and preferred location for living or dying (Gramling et al., 2015). Similar research concluded that communication strategies for goals-of-care conversations include describing the signs that indicate a transition in care is approaching and explicitly stating when a patient or family stated goal is not clinically feasible (Norton et al., 2013).

However, findings from this study depict a different scope and role for APs, demonstrating an emphasis on patient understanding and family support rather than delivering or discussing prognosis. The content of goals-of-care conversations for APs may vary depending on the health-care team, clinical setting, and patient/family understanding. The minimal presence of team in goals-of-care communication may indicate the need to clarify how a team can address goals of care and improve team function when serving patients and families. Future work is needed to determine team-based preferences for the AP’s role in these conversations.

One of the limitations of this study is the sample’s presence at an educational training session for end-of-life nursing practice. The providers attending had experience and knowledge of goals-of-care communication not typical of other nursing specialists and practitioners. Perspectives from nurses in different clinical settings should be studied in future research.

The lack of a formal review process to evaluate performance in goals-of-care conversations further impedes professional growth in the area of communication skills development (McCorkle et al., 2012). Future studies need to evaluate interventions to improve communication and shared decision-making (Kryworuchko, Hill, Murray, Stacey, & Fergusson, 2013). Further research on option grids, which are a tool to help patients compare alternative treatment options, showed early promise for patient engagement in decision-making in partnership with providers (Elwyn et al., 2013a).

## CONCLUSION

This study demonstrates that nurses perceive primary communication in goals of care to focus on the task of assessing patient understanding. Nurses reported that their most frequent concerns over goals-of-care communication were the function, structure, and process of the care team. Findings demonstrate that preparation to function as a team in the context of goals-of-care communication is a barrier for nurse participants, and they report a need to move goals of care upstream in the care provided to patients and their families. Future research should explore patient and family expectations of nurses and teams during goals-of-care conversations to shape education and curricular development for the next wave of palliative care providers.

The study has important implications for APs in oncology. These providers are the ones who discuss initial diagnosis and treatment options as well as prognosis with patients. They are also facilitators of advanced-care planning, team meetings, and family conferences. Each of these roles has a strong impact on quality patient care and family support.

## References

[1] Adams J A, Bailey, Jr. Donald E, Anderson R A, Docherty S L (2011). Nursing roles and strategies in end-of-life decision making in acute care: A systematic review of the literature.. *Nurse Research and Practice*.

[2] Bakitas Marie, Kryworuchko Jennifer, Matlock Dan D, Volandes Angelo E (2011). Palliative medicine and decision science: the critical need for a shared agenda to foster informed patient choice in serious illness.. *Journal of palliative medicine*.

[3] Beckstrand Renea L, Collette Joan, Callister Lynn, Luthy Karlen E (2012). Oncology nurses’ obstacles and supportive behaviors in end-of-life care: providing vital family care.. *Oncology nursing forum*.

[4] Bélanger Emmanuelle, Rodríguez Charo, Groleau Danielle (2011). Shared decision-making in palliative care: a systematic mixed studies review using narrative synthesis.. *Palliative medicine*.

[5] Bernacki Rachelle E, Block Susan D (2014). Communication about serious illness care goals: a review and synthesis of best practices.. *JAMA internal medicine*.

[6] Charles C, Gafni A, Whelan T (1997). Shared decision-making in the medical encounter: what does it mean? (or it takes at least two to tango).. *Social science & medicine (1982)*.

[7] Elo Satu, Kyngäs Helvi (2008). The qualitative content analysis process.. *Journal of advanced nursing*.

[8] Elwyn G, Frosch D, Volandes A E, Edwards A, Montori V M (2010). Investing in deliberation: A definition and classification of decision support interventions for people facing difficult health decisions.. * Medical Decision Making*.

[9] Elwyn Glyn, Lloyd Amy, Joseph-Williams Natalie, Cording Emma, Thomson Richard, Durand Marie-Anne, Edwards Adrian (2013). Option Grids: shared decision making made easier.. *Patient education and counseling*.

[10] Elwyn G, Scholl I, Tietbohl C, Mann M, Edwards A G, Clay C, Frosch D L (2013b). “Many miles to go...”: A systematic review of the implementation of patient decision support interventions into routine clinical practice.. *BMC Medical Informatics and Decision Making*.

[11] Goldsmith Joy, Ferrell Betty, Wittenberg-Lyles Elaine, Ragan Sandra L (2013). Palliative care communication in oncology nursing.. *Clinical journal of oncology nursing*.

[12] Gramling Robert, Sanders Mechelle, Ladwig Susan, Norton Sally A, Epstein Ronald, Alexander Stewart C (2015). Goal Communication in Palliative Care Decision-Making Consultations.. *Journal of pain and symptom management*.

[13] Kawachi I, Kennedy B P (1997). Health and social cohesion: why care about income inequality?. *BMJ (Clinical research ed.)*.

[14] Klarare Anna, Hagelin Carina Lundh, Fürst Carl Johan, Fossum Bjöörn (2013). Team interactions in specialized palliative care teams: a qualitative study.. *Journal of palliative medicine*.

[15] Kryworuchko Jennifer, Hill Elina, Murray Mary Ann, Stacey Dawn, Fergusson Dean A (2013). Interventions for shared decision-making about life support in the intensive care unit: a systematic review.. *Worldviews on evidence-based nursing / Sigma Theta Tau International, Honor Society of Nursing*.

[16] Malloy P, Virani R, Kelly K, Munevar C (2010). Beyond bad news: Communication skills of nurses in palliative care.. *Journal of Hospice & Palliative Nursing*.

[17] Martin Beth, Koesel Niki (2010). Nurses’ role in clarifying goals in the intensive care unit.. *Critical care nurse*.

[18] McCaffery, K J, Holmes-Rovner M, Smith S K, Rovner D, Nutbeam D, Clayman M L, Sheridan S L (2013). Addressing health literacy in patient decision aids.. *BMC Medical Informatics and Decision Making*.

[19] McCorkle Ruth, Engelking Constance, Knobf M Tish, Lazenby Mark, Davies Marianne, Sipples Rebecca, Ercolano Ellyn, Lyons Catherine (2012). Transition to a new cancer care delivery system: opportunity for empowerment of the role of the advanced practice provider.. *Journal of the advanced practitioner in oncology*.

[20] Moir Cheryl, Roberts Renee, Martz Kim, Perry Judith, Tivis Laura J (2015). Communicating with patients and their families about palliative and end-of-life care: comfort and educational needs of nurses.. *International journal of palliative nursing*.

[21] Moumjid N, Gafni A, Brémond A, Carrère M O (2007). Shared decision making in the medical encounter: Are we all talking about the same thing?. *Medical Decision Making*.

[22] National Consensus Project for Quality Palliative Care. (2013). * Clinical Practice Guidelines for Quality Palliative Care (3rd Ed.).*.

[23] Norton Sally A, Metzger Maureen, DeLuca Jane, Alexander Stewart C, Quill Timothy E, Gramling Robert (2013). Palliative care communication: linking patients’ prognoses, values, and goals of care.. *Research in nursing & health*.

[24] The Patient Protection and Affordable Care Act, 42 U.S.C. § 18001. (2010). https://www.gpo.gov/fdsys/pkg/PLAW-111publ148/html/PLAW-111publ148.htm.

[25] Svarovsky Therese (2013). Having Difficult Conversations: The Advanced Practitioner’s Role.. *Journal of the advanced practitioner in oncology*.

[26] Tamburro Robert F, Shaffer Michele L, Hahnlen Nicole C, Felker Paul, Ceneviva Gary D (2011). Care goals and decisions for children referred to a pediatric palliative care program.. *Journal of palliative medicine*.

[27] Wu J J, Downar J, Fowler R A, Lamontagne F, Ma I W, Jayaraman D, Canadian Researchers at the End of Life Network (2015). Barriers to goals of care discussions with seriously ill hospitalized patients and their families: A multicenter survey of clinicians.. *JAMA Internal Medicine*.

[28] You John J, Downar James, Fowler Robert A, Lamontagne François, Ma Irene W Y, Jayaraman Dev, Kryworuchko Jennifer, Strachan Patricia H, Ilan Roy, Nijjar Aman P, Neary John, Shik John, Brazil Kevin, Patel Amen, Wiebe Kim, Albert Martin, Palepu Anita, Nouvet Elysée, des Ordons Amanda Roze, Sharma Nishan, Abdul-Razzak Amane, Jiang Xuran, Day Andrew, Heyland Daren K (2015). Barriers to goals of care discussions with seriously ill hospitalized patients and their families: a multicenter survey of clinicians.. *JAMA internal medicine*.

